# Interactions of antidepressants with concomitant medications—safety of complex therapies in multimorbidities

**DOI:** 10.1007/s43440-024-00611-7

**Published:** 2024-07-16

**Authors:** Anna Dołoto, Ewelina Bąk, Gabriela Batóg, Iwona Piątkowska-Chmiel, Mariola Herbet

**Affiliations:** Chair and Department of Toxicology, Faculty of Pharmacy, Medical University of Lublin, Jaczewskiego 8B Street, 20-090 Lublin, Poland

**Keywords:** Depression, Antidepressants, Drug-drug interactions, Polypharmacotherapy

## Abstract

Depression is the fourth most serious disease in the world. Left untreated, it is a cause of suicide attempts, emergence or exacerbation worsening of serious diseases, bodily and mental disorders, as well as increased risk of cardiovascular diseases, stroke, diabetes, and obesity, as well as endocrine and neurological diseases. Frequent coexistence of depression and other diseases requires the simultaneous use of several drugs from different therapeutic groups, which very often interact and intensify comorbidities, sometimes unrelated mechanisms. Sufficient awareness of potential drug interactions is critical in clinical practice, as it allows both to avoid disruption of proper pharmacotherapy and achieve substantive results. Therefore, this review aims to analyze the interactions of antidepressants with other concomitant medications. Against the backdrop of experimental research and a thorough analysis of the up-to-date literature, the authors discuss in detail the mechanisms and effects of action of individual drug interactions and adaptogens, including the latest antidepressants.

## Introduction

According to the World Health Organization, depression is the fourth most serious disease in the world and one of the leading causes of suicide, appearing more and more often in all age groups. It is predicted that 2030 it will be the most common worldwide spread disorder [1]. Although the leading causes of depression remain vastly unexplored, it appears to be linked to various global circumstances, such as terrorism, pandemics, economic crises, and war [2]. In many cases, depression is seldom accompanied by other disorders, such as cardiovascular diseases [3], Parkinson's disease [4], epilepsy [5], and polycystic ovary syndrome (PCOS) [6]. Up-to-date studies indicate the coexistence of depression with somatic diseases. People suffering from depression are also exposed to cardiovascular diseases, stroke, diabetes, and obesity [7]. Such correlation is evident in older patients [8], while the depression of young people appears to be linked to thyroid diseases, type 2 diabetes, other endocrine diseases, as well as neurological diseases [9]. Untreated depression leads to suicide attempts [A1], the emergence or worsening of severe diseases, bodily and mental disorders, as well as numerous social and economic problems. Against this backdrop, it is agreed that major depression requires pharmacological treatment, which is a long-term process that should be continued for 6–12 months. In about 50–70% of cases, the first used antidepressant indicates positive results. However, the remaining patients require additional or alternative medication due to the ineffectiveness of the first attempt drug or experienced side effects [10]. Many patients also self-medicate, taking increasingly advertised and readily available adaptogens.

Simultaneous treatment of depression and other diseases leads to the use of several drugs from different therapeutic groups [8, 9]. At the same time, antidepressants are known for their everyday interaction with other medications and causing hurt comorbidities (drug-disease interactions), posing a threat to the health and life of patients. Therefore, one of the most severe problems of modern pharmacotherapy is the selection of appropriate therapy in patients treated for various diseases that require multidrug treatment [8, 9]. This applies to unfavorable interactions between concurrently used drugs and the resulting clinical effects. These may occur through unrelated mechanisms. Antidepressants can interact with other medications, primarily through pharmacokinetic processes affecting their absorption, distribution, metabolism, and excretion, which may alter their effectiveness or lead to toxicity. These interactions also involve pharmacodynamic mechanisms, such as synergism or antagonism, impacting drug efficacy and potential toxicity. There is a complex and not yet fully understood relationship between antidepressants and the drug efflux pump. This compound may significantly affect the therapeutic effectiveness of antidepressant treatment. P-glycoprotein plays an essential role in the functioning of the blood–brain barrier by selectively extruding drugs, thereby limiting the ability of its substrates to reach the brain [11]. On the one hand, this protein may limit the uptake of antidepressants into the brain, weakening antidepressant therapies. Drugs or other substances that are inhibitors or inducers of P-glycoprotein may also modulate the pharmacological activity of antidepressants. If other drugs simultaneously inhibit the transport protein, the penetration of the substrate drug increases, and its effect is enhanced. In case of excessive penetration of drugs through the blood–brain barrier, the side effects of antidepressants may be intensified, including serotonin syndrome, neurotoxicity, etc. However, controlled co-administration of transport protein inhibitors with antidepressants in antidepressant-resistant patients may represent a new therapeutic approach for the treatment of depression. In turn, drugs or other substances that are P-glycoprotein inducers may block the penetration of antidepressants into the central nervous system. They may weaken or even eliminate the antidepressant effect [12] P-glycoprotein in vitro, which may play a role in their therapeutic effect.

In clinical practice, a clear understanding of potential drug interactions is essential to avoid those that could interfere with the proper course of the pharmacotherapy process, as well as to use some interactions to benefit the patients. The currently available scientific literature does not provide a comprehensive summary of the interactions of all groups of antidepressants with other concomitantly taken medications or foods. The available literature lacks comprehensive reports on the interaction of antidepressants with other drugs. Therefore, the aim of this review is a thorough analysis of the interaction of antidepressants, taking into account the mechanisms of formation and side effects.

### Interactions of tricyclic antidepressants (TCAs)

Tricyclic antidepressants (TCAs), introduced for the treatment of depression in the 1950s, are now rarely used due to numerous side effects [13]. The mechanism of action of these drugs is blocking the reuptake of neurotransmitters—norepinephrine and serotonin by nerve cells, which leads to an increase in the concentration of these transmitters in synaptic clefts. They also act as antagonists of muscarinic, histamine H1, and postsynaptic α1 and α2 adrenergic receptors [14]. TCAs interactions include primarily the risk of serotonin syndrome when taken together with monoamine oxidase inhibitors (MAOI) and selective serotonin reuptake inhibitor (SSRI) medications [15] (Table 1). There is also a risk of serotonin syndrome when tricyclic antidepressants are taken concomitantly with linezolid, tramadol, ondansetron, lithium compounds, fentanyl, buspirone, and preparations containing St. John's wort [16]. The serotonin syndrome is a phenomenon that directly threatens human life. It includes autonomic overactivity, a change in the patient's mental state, hypomania, normal neuromuscular tension, restlessness, confusion, myoclonus, and an increase in the patient's body temperature [17].

**Table 1:** Interactions of TCAs with other medications drug classes and food

Interactions of TCAs with other drugs classes			
Drug	Interacting substance	Result of interaction	References
TCAs	Ezyme CYP1A2 inhibitors: Cimetidine Tacrolimus Warfarin Antipyrine Verapamil Theophylline Diltiazem Ciprofloksacin Methoxsalen Norfloksacin Mexiletine Norethindrone Caffeine Nalidixic acid Citalopram Miodarone Clarithromycin Fluoxetine Erythromycin Melatonin Ethinylestriadol Fluvoxamine Isoniazid Troleandomycin Paroxetine Ticlopidine Tacrine Oral anticonceptives Grape juice Cumin	Increased risk of side effects of TCAs	[15, 19] [16]
	Enzyme CYP 1A2 inductors: Carbamazepine Omeprazole Primidone Barbiturates Rifampicin	Shorten the effect of the TCAs increase the effect of that batch of drugs that have undergone biotransformation in the body to their active metabolites	
TCAs	SSRI MAOI Linezolid Tramadol Ondasetron Lithium compounds Fentanyl Buspirone St. John’s wort	Serotonin syndrome	[15–18, 21–26]
	Drugs that affect cardiac conduction Antiarrhythmic drugs diuretic Phenothiazine drugs Antipsychotic Proconvulsant	Decrease the seizure threshold, loss of control over epileptic attacks	
	Anticholinergic Sedative drugs	Dxacerbate such side effects as confusion, constipation and bowel problems and visual disturbances	
	Atropine Scopolamine	Delirium	
	Antimuscarinic substances peripheral Central anticholinergic	Urinary retention, memory impairment, confusion, blurred vision, intestinal obstruction, dry mouth, glaucoma and constipation	
	CNS depressants	Deterioration of psychomotor performance and may also adversely affect cognitive and psychomotor functions	
	Epinephrine	Promoting an increase in blood pressure and cardiac arrhythmias	
	Cholestyramine	Significant decrease in the concentration of TLPD in the body of patients and the subsequent lack of pharmacological effect	
	Oral hormonal therapy Estrogens	Incidents of akathisia and a decrease in the potential and exacerbation of toxicity of TLPD	
	Enalapril	Incidents of accommodative dysfunction and blurred vision	
	Fluoroquinolones	Significant increase in TLPD and subsequent patient cardiac dysfunction	
	Halothane	Increased risk of hyperthermia	
	Antidiabetic drugs	Subsequent incidents of hypoglycemia and decrease in hypoglycemic potential	
	Levothyroxine	Incidents of tachycardia	
	Melatonin Methadone Quetiapine	Pathological increase in the patient's sedation	
	Pancuronium	The possibility of hyperthermia	
	Coffee or tea	Reducing effectiveness of drugs	

Cytochrome CYPA1A2 plays a significant role in the metabolism of drugs in this group [18]. Concurrent administration of TCAs and medicines or foods that are inhibitors or inducers of the CYPA1A2 isoenzyme may impair the metabolism of antidepressants. The use of isoenzyme inhibitors may increase the plasma concentration of tricyclic antidepressants and the intensification of their side effects. In contrast, enzyme inducers may weaken their impact and increase the concentration of their active metabolites [16]. TCA should be used with great caution in people who take drugs that affect cardiac conduction, antiarrhythmic drugs, diuretics, and phenothiazine drugs.

Moreover, individuals taking these drugs and antipsychotic and proconvulsant substances concurrently should also receive special monitoring and caution due to the ability of tricyclic antidepressants to decrease the seizure threshold [16]. In the case of maprotiline and clomipramine, the occurrence of this effect may lead to loss of control over epileptic seizures in people suffering from this condition [19]. Moreover, nortriptyline and imipramine increase the concentration of phenytoin in the body of epilepsy patients, which is related to the inhibitory effect of these antidepressants on the microsomal subunit of cytochromes CYP2C19 and CYP2D6. These processes lead, among others, to inhibit the reaction, resulting in p-hydroxylated phenytoin, indicating that both antidepressants act as competitive substrates for phenytoin [20].

As a result of the combination of TCAs with antimuscarinic substances, peripheral and central anticholinergic effects may be intensified, which may result in urinary retention, memory impairment, confusion, blurred vision, intestinal obstruction, dry mouth, glaucoma, and constipation {Citation} [16, 18]. The use of a combination of the tricyclic antidepressant amitriptyline and L-DOPA indicates a favorable interaction between the two drugs, which carries an increased antiparkinsonian effect compared to conventional therapy with L-DOPA alone [21].

Moreover, TCAs used in combination with central nervous system (CNS) depressants may lead to deterioration of psychomotor performance and may also adversely affect cognitive and psychomotor functions [22]. The combination of TCAs with antipsychotic drugs may lead to toxic concentrations of antidepressants in the body, which is related to the inhibition of the cytochrome subunit CYP2D6 [22]. TCAs block the reuptake of antihypertensive drugs by adrenergic neurons, which leads to the absence or weakening of the effects of drugs such as bretylium, guanethidine, betanidine, and debrisoquine. In addition, their interaction with α2 adrenergic receptors can attenuate the effect of drugs such as methyldopa and clonidine. On the other hand, drugs belonging to the calcium antagonist group, such as diltiazem and verapamil, enhance the pharmacological effect and overall bioavailability of imipramine. This effect results from these drugs competing as substrates with the cytochrome subunit CYP3A4 [22].

Studies have shown that the antifungal drug ketoconazole blocks the demethylation of imipramine while not affecting desipramine hydroxylation [22]. An increase in nortriptyline concentration was observed with simultaneous administration of fluconazole [22]. The concurrent use of warfarin and amitriptyline may cause hemorrhagic reactions [22]. Concomitant intake of yohimbine and TCAs can lead to hypertension. The discussed effect is even more potent in patients who already have hypertension [23]. Reducing the effectiveness of antidepressant therapy and possible intensification of adverse side effects may occur when consuming herbs such as Kava kava or the St. John's Wort [24]. In addition, laxative herbs such as Plantago spp. Plants, konjac, or guar gum can delay gastric emptying and diminish drug absorption [25]. Additionally, the cytochrome CYP1A2 subunit responsible for metabolizing procarcinogens contained in cigarette smoke can be induced by these substances, thereby altering the metabolism of tricyclic antidepressants [26]. Foods that affect the function of this enzyme include grilled meat, broccoli, tobacco, and brussels sprouts [24].

### Interactions of SSRIs

Selective serotonin reuptake inhibitors (SSRIs) enhance the level of neurotransmitters such as serotonin, dopamine, and norepinephrine. SSRIs selectively affect the serotonergic system by inhibiting serotonin reuptake, which improves serotonin levels in the space between neurons and prolongs their action in the brain. An increase in serotonin levels occurs approximately two weeks after starting SSRI therapy [27]. The cytochrome P450 in the liver metabolizes SSRIs. This group of antidepressants can inhibit some CYP450 enzymes; thereby, drug-drug interactions can occur (Table 2).

**Table 2:** Effects of selected SSRI drugs on isoenzyme inhibition

Effects of selected SSRI drugs on isoenzyme inihibition						References
	CYP2D6	CYP2C9	CYP2C19	CYP3A4	CYP1A2	
Fluoxetine/norfluoxetine	Dominant	Limited	Moderate	Moderate		[3, 28–31]
Paroxetine	Decided					
Sertraline	Dose-epended					
Fluvoxamine		Limited	Strong	Limited	Strong	
Citalopram	Feeble		Irrelevant	Irrelevant	Irrelevant	
Citalopram	Feeble		Irrelevant	Irrelevant	Irrelevant	

Based on available studies, severe depression symptoms can involve 45% of patients who have coronary artery disease (CAD), and circa 20% of them can have all the parameters of a full depressive episode. Woroń et al. analyzed 66 cases. Most antidepressants and cardiac drug interactions involved SSRIs. Bradycardia was the side effect that occurred the most often after the combination of SSRI and metoprolol or propranolol. In one case, the consequence of a combination of fluoxetine and propranolol was sudden cardiac arrest. Antidepressants inhibit the activity of CYP2D6—the enzyme that metabolizes metoprolol and propranolol the most [3]. Of the SSRIs, venlafaxine and sertraline have the least severe side effects in combination with metoprolol and propranolol. In case of use of other drugs of SSRI or serotonin and norepinephrine reuptake inhibitors (SNRI) group, metoprolol, and propranolol should be exchanged into nebivolol or carvedilol because they have a lower risk of interactions [3]. Except for the above study, there are available studies describing the case of a 63 years woman who had anterograde block after a combination of metoprolol and paroxetine [28] and the case of a 53 years man who had the same side effect after a combination of propranolol and fluoxetine [29]. A similar mechanism to that observed with SSRIs and β-blockers concerns propafenone and β-blockers. Propafenone is metabolized by CYP2D and 3A4 [3]. Case reports have documented instances of worsened propafenone tolerance (bradycardia, prolongation of QT) following its combination with citalopram [30]. Another group in which were observed side effects was a combination of SSRIs and calcium channel blockers. Side effects of calcium channel blockers increased, with cases of acute renal failure (after the combination of fluoxetine and amlodipine) and cases of myalgia, polyuria, elevated levels of transaminases (combination of SSRIs and lercanidipine), and one case of severe bradycardia (combination of fluvoxamine and diltiazem) [3]. The literature also describes the occurrence of edema, headaches, and weight gain after combining fluoxetine with verapamil [31]. These interactions between SSRIs and calcium channel blockers appear primarily pharmacokinetic, involving competition for the same CYP450 isoenzyme or inhibiting the isoenzyme responsible for metabolizing calcium channel blockers SSRIs [3].

SSRIs block the reuptake of serotonin from the platelets. As a result, the inhibitory of platelet aggregation occurs, and it can cause an increased risk of bleeding [32] in patients who take other drugs to decrease coagulation. This risk concerns patients who take antiplatelet drugs (acetylsalicylic acid, clopidogrel, ticagrelor), as well as patients taking anticoagulants, which have another mechanism of action (warfarin, dabigatran, rivaroxaban, acenocoumarol, apixaban, heparin). During the simultaneous treatment of SSRIs and warfarin, the additional hemorrhagic risk may be connected with inhibiting warfarin metabolism (CYP 450: 2C9, 2C19, 3A4) and its increased level in the blood. In most cases, this interaction may occur during the treatment of fluvoxamine, fluoxetine, and paroxetine, while minor cases may occur with sertraline. Moreover, fluvoxamine and fluoxetine block CYP2C19 and consequently reduce the antiplatelet effects of clopidogrel by inhibiting clopidogrel's transformation into an active metabolite. According to Woroń et al., the combination of anticoagulants, such as acenocoumarol, apixaban, acetylsalicylic acid, rivaroxaban, and SSRIs, such as citalopram, escitalopram, fluoxetine, fluvoxamine, sertraline, and venlafaxine may increase the risk of hemorrhagic complication syndrome, including upper or lower gastrointestinal bleeding [33]. An increased incidence of hemorrhagic complications is also observed with concomitant use of SSRIs with glucocorticosteroids and non-steroidal anti-inflammatory drugs [33]. Treatment of vitamin K antagonists (VKA) and SSRIs simultaneously may be associated with an increased risk of bleeding. There are three known mechanisms which may correspond to the above occurrence. Firstly, it is due to the inhibition of serotonin reuptake in platelets by SSRIs, which worsens their function and may cause an increased risk of bleeding. Secondly, SSRIs may increase the secretion of gastric acid, which may increase the risk of peptic ulcer disease and bleeding from the upper gastrointestinal tract. Third, inhibition of CYP2C9 may lead to a high international normalized ratio (INR) and an increased risk of bleeding [34]. SSRIs taken simultaneously with high doses of loop diuretics and thiazides may lead to hyponatremia, which is particularly dangerous in elderly patients [3]. Hyponatremia may occur as a result of thiazide diuretics with citalopram or escitalopram, as well as diuretics with sertraline fluoxetine, and fluvoxamine [33]. The mechanism of this side effect may be associated with the risk of summing hyponatremia, which stems from the action of the diuretic and the risk of induction of the syndrome of inappropriate antidiuretic hormone hypersecretion connected with blockade of serotonin reuptake [3]. Esomeprazol and omeprazole inhibit CYP 2C19, leading to the increased blood concentration of citalopram and escitalopram and subsequently amplifying their adverse effects. Moreover, esomeprasol inhibits CYP 3A4, potentially increasing sertraline concentration and its side effects. Fluoxetine inhibits isoenzymes CYP 2C9, 2C19, and 3A4, leading to increased lansoprazole concentration and the risk of side effects. A similar effect is observed with fluvoxamine, which inhibits 2C19 and 3A4 [33]. Concomitant use of itraconazole, fluconazole, ketoconazole, and miconazole with venlafaxine can increase its side effects by inhibiting CYP3A4, leading to elevated venlafaxine blood concentrations. Fluconazole’s inhibition of CYP2C19 and 3A4 may heighten adverse reactions to citalopram and escitalopram [33]. Citalopram and escitalopram can prolong the QT interval on electrocardiogram (ECG) by affecting potassium channels, potentially causing ventricular arrhythmia when co-administered with anti-arrhythmic drugs like amiodarone, propafenone, and sotalol. Statins, especially simvastatin, atorvastatin, and lovastatin, may interact with SSRIs by inhibiting CYP3A4, increasing the risk of statin-related side effects such as myopathy, myalgia, or rhabdomyolysis, particularly when combined with fluvoxamine [3, 35]. Fluoxetine strongly inhibits the CYP 3A4 isoenzyme and, as a result, may increase aripiprazole concentrations by up to 45%, thereby increasing the risk of adverse effects. Fluvoxamine inhibits CYP 2D6 and 3A4, resulting in an increase in aripiprazole concentrations and the need for a 50% dose reduction [33]. Detailed interactions of SSRIs are presented in Tables 3 and 4.

**Table 3:** Interactions of SSRIs with other medications drug classes

Interactions of SSRIs with other drug classes			
Drug	Interacting substance	Result of interaction	References
Citalopram/escitalopram	Itopride	Risk of late diskinesis	[3, 32–34]
	Clonidine	Cases of hyponatremia and sedation	
	Lamotrigine	Cases of myoclonus	
	Drugs that prolong QT interval	Increased risk of cardiac conduction and rhythm disturbances, including ventricular arrhythmias	
	Levomepromazine	Increased risk of side effects of citalopram or escitalopram	
	Melatonin	exacerbation of its effects and increased risk of side effects	
	Rasagiline	Greater risk of disturbance of consciousness	
	Rifampicine	Decreased in efficacy of citalopram or escitalopram	
	Risperidon	Cases of ventricular arrhythmias	
	Activated carbon	Decreased absorption of drugs from the gastrointestinal tract	
Fluoxetine	Amlodipine	Cases of acute renal failure and peripheral oedema	
	Bupropion	Cases of unconsciousness	
	Buspirone	Reduced anti-anxiety effects, cases of akathisia, dystonia	
	Phenytoin	Increased side effects and toxicity and reduced efficacy of fluoxetine	
	Lamotrigine	Cases of delirium	
	Lercanidipine	Cases of hypotention	
	Lurasidone	Cases of pharyngeal dystonia	
	Metoprolol	Cases of bradycardia and conduction blocks	
	Nifedipine Verapamil	Cases of oedema, nausea, hot flashes	
	Olanzapine Propafenone Haloperidol Iloperidol Alprazolam Atomoxetine Cyclosporine Diazepam Valproate	Increased risk of side effects	
	Propranolol	Cases of bradycardia, conduction blocks and cardiac arrest	
	Selegiline	Increased risk of ataxia toxicity, increased blood pressure	
	Tamoxifen	Decreased efficacy	
	Tramadol	Decreased analgesic effect	
	Carbamazepine	Increased risk of side effects, hyponatremia and cases of blurred vision, tremors	
	Clarithromycin	Delirium	
	Zolpidem	Increased risk of hallucinations	
Fluvoxamine	Agomelatine Phenytoin Haloperidol Clozapine Tianisidine Alprazolam Asenapine Buspirone Cyclosporine Diazepam Diltiazem Fexofenadine Phenothiazine Caffeine Quetiapine	Increased risk of side effects	
	Celecoxib	Cases of agitation and delirium	
	Carbamazepine	Increased side effects, decreased efficacy of fluvoxamine, risk of hyponatremia	
	Levomepromazine	Cases of convulsions	
	Lidocaine	Increased toxicity	
	Olanzapine	Increased risk of side effects, including convulsions, sedation, hypotonia, tachycardia, hepatotoxicity	
	Piracetam	Decreased its excretion, resulting in increased blood concentrations	
	Theophylline	Increased toxicity	
	Topiramate	Cases of tremors and myoclonus	
	Zolpidem	Increased risk of hallucinations and memory impairment	
Sertraline	Amlodipine	cases of headache and oedema	
	Cisapride	cardiac arrhythmias	
	Phenytoin	Decreased sertraline concentration and efficacy, increased risk of phenytoin toxicity	
	Carbamazepine	Decreased sertraline concentration and efficacy, increased carbamazepine concentrations and side effects	
	Lamotrigine	Cases of fatigue, sedation, cognitive impairment	
	Lercanidipine	Cases of myalgia and polyuria	
	Oxycodone	Cases of hallucinations and tremors	
Venlafaxine	Amiodarone Bupropion Celecoxib Diphenhydramine Duloxetine Erythromycin Phenothiazines Haloperidol Indinavir Clarithromycin Mexiletine Methadone Nilotinib Pindolol Risperidone Ritonavir Terbinafine Ticlopidine	Increased concentration of vanlafaxine in blood and risk of side effects	
	Amoxicillin with clavulanic acid	Cases of serotonin syndrome	
	Clozapine	Increased concentration of vanlafaxine in blood and risk of side effects, increased risk of tachycardia and blood pressure changes	
	Metoprolol	Decreased blood pressure lowering efficacy of metoprolol, increased venlafaxine concentrations and side effects	
	Piracetam	Decreased excretion of piracetam, resulting in an increase in blood concentrations of the drug	
	Propafenone	Cases of psychosis, bradycardia, dizziness, visual disturbances	
	Propranolol	Cases of increased urinary urgency and increased muscle tone

**Table 4:** Interactions of SSRI drugs with plant substances

Interactions of selected SSRI drugs with plant substances			
Drug	Interacting substance (herb)	Result of interaction	References
Citalopram, escitalopram, fluoxetine, fluvoxamine, venlafaxine, sertraline	*Ginkgo biloba* *Milk thistle* *Rhodiola rosea*	Increased risk of bleeding Coughing, ejaculatory disorders Myalgia, cardiac arrhytmias	[40–43]
Citalopram or escitalopram, fluoxetine, fluvoxamine	*Panax ginseng*	Ejaculatory disorders, priapism and an increased risk of serotonergic syndrome	
Fluoxetine	*Bacopa monnieri* *Purple coneflower* *Rhiodola rosea* and additionally duloxetine	Increased risk of serotonergic syndrome Sudden hair loss and priapism Cases of diarrhoea, jaundice and hepatotoxicity	
Sertraline	*Milk thistle* and agomelatine *Panax ginseng* *Starfruit*	Hepatotoxicity Chest pain, tachycardia, cortical arrhthmias Increased risk of serotonine syndrome, cases of bruxism, headaches	
Venlafaxine	*Jujuba* *Panax ginseng*	Increased risk of serotoninergic syndrome Increased the risk of hyperhidrosis and itching of the skin	

### Interactions of tetracyclic antidepressants

Maprotiline is a drug included in the group of quadricyclics used to treat depression, which very strongly inhibits the reuptake of norepinephrine, thereby increasing its concentration in the synaptic gap [36, 37]. Available literature also reports that it may prove effective in eradicating *Francisella* infection as an antiviral agent through its ability to decrease the virulence index of IgIC [38]. Moreover, due to its anabolic nature, it can be used by people who have anorexia, underweight, and those in recovery [39]. The cytochrome CYP2D6 subunit mainly metabolizes maprotiline, and concomitant administration of inhibitors or inductors carries the risk of drug adverse effects [40] (Table 5) [41–43].

**Table 5:** Interactions of tetracyclic antidepressants with other medications. drug classes

Interactions of tetracyclic antidepressants with with other drug classes			References
Drug	Interacting substance	Result of interaction	
Tetracyclic antidepressants (maprotiline)	Ezyme CYP1A2 inhibitors: Diltiazem Celecoxib Moclobemide Bupropion Metoclopramide Encapone Yohimbine Methadone Codeine Dextropropoxyphene Esipramine Imipramine Clomipramine Lobeline Metoclopramide Tricyclic antidepressants Risperidone	Elevation of the plasma level of tetracyclic antidepressants, increased cholinergic effects	[44–46]
	Enzyme CYP2D6 inductors: Griseofulvin Rifabutin Rifampicin Sulfadimidine Nafcillin Troglitazone Ethosuximide Carbamazepine Phenytoin Phenobarbital Nevirapine Nelfinavir Progesterone Refexib Phenylbutazone Dexamethasone	Shorten the effect of the tetracyclic antidepressants, increase the effect of that batch of drugs that have undergone biotransformation in the body to their active metabolites	
Tetracyclic antidepressants (maprotiline)	Dextromethorphan	Development of cardiomyopathy	
	Ritonavir Fluconazole	Orthostatic hypotension	
	Phenytoin	Ventricular tachycardia	
	Betanidine	Increased lack of control over normal blood pressure	
	Reboxetine	Serotonin syndrome	
	Desmopressin	Increase in water retention in the body, Pathological state of hyponatremia	
	Verapamil	Development of severe heart failure	

A case of cardiogenic shock associated with Takotsubo cardiomyopathy has also been reported during the concomitant administration of maprotiline and dextromethorphan. The development of cardiomyopathy was closely related to the presence of serotonergic interactions between the two drugs, which resulted later in the occurrence of serotonin syndrome. A woman aged 65 was treated with maprotiline due to the presence of depressive symptoms. The initial dose was 10 mg/24 h and was increased to 30 mg/24 h. Later, due to bronchitis, dextromethorphan and amoxicillin were included in her therapy. Three days after the list of medications was expanded, the woman developed symptoms such as hemoptysis, shock, severe dyspnoea, and acute agitation. An X-ray taken after admission showed lower lobe consolidation, ST-segment elevation, and pulmonary congestion. Other tests showed elevated levels of creatine kinase-MB and creatine kinase, basal hyperkinesis, apical akinesis, apical ballooning, and an elevated c-reactive protein (CRP) index. After intubating the patient and administering norepinephrine and dobutamine, she was diagnosed with Takotsubo cardiomyopathy. After taking the necessary measures, she was diagnosed with serotonin syndrome the next day. It was decided to exclude maprotiline from therapy and include cyproheptadine and midazolam. After this regimen, the patient’s temperature dropped, and hemodynamic indicators were regulated to safe levels [44].

Episodes of ventricular tachycardia have also been documented to affect the development of severe heart failure when maprotiline was combined with drugs used for epileptic seizures. The woman, aged 69, had been on therapy with glycoside and verapamil—antiarrhythmic drugs—for 13 years to normalize her atrial fibrillation and on long-term maprotiline therapy to treat her depression. The day before the onset of adverse and chewing-threatening symptoms, the woman used phenytoin to normalize the epileptic seizures she was experiencing. After the patient was admitted to the ward and the necessary examinations were performed, she was shown to have torsade de pointes tachycardia, reduced ejection fraction, coronary artery obstruction, left ventricular dilatation, and atherosclerotic plaques. After appropriate interventions, including defibrillation, the patient was administered xylocaine and magnesium, and maprotiline and antiarrhythmic medications were discontinued. After the implementation of these treatments, the characteristic tachycardia stopped, and the QT interval shortened. The occurrence of these symptoms results from possible drug interactions between antidepressants, arrhythmogenic drugs, and existing diseases. This case illustrates that the use of multidrug therapy with drugs of a narrow therapeutic range should be strictly controlled and subject to continuous observation [45]. Concurrent use of maprotiline and drugs that block adrenergic neurons can increase the risk of losing control over normal blood pressure. This interaction arises from competition between the two drug types for uptake into vesicles located in sympathetic neurons. In one study, patient subjects experienced a notable rise in blood pressure after receiving two 25 mg of maprotiline. Other clinical cases reported significant blood pressure dysregulation in patients receiving maprotiline doses ranging from 25 to 75 mg thrice daily. The introduction of maprotiline into the therapy of the time in four patients disrupted normal blood pressure in one patient [46].

Studies with animals also show evidence of interactions between maprotiline and drugs with central nervous system depressant effects [46]. The occurrence of life-threatening serotonin syndrome has also been described with the concomitant use of venlafaxine 300 mg/24 h, reboxetine 8 mg/24 h, and maprotiline 150 mg/24 h. A man undergoing such therapy developed serotonin syndrome. He experienced symptoms such as partial confusion, mutism, confusion, negativism, non-Parkinsonian rigidity, tremors in the upper extremities, episodes of psychomotor agitation, excessive sweating, hyper-responsiveness to bone-tendon reflexes, and hyperreflexia. The patient’s blood pressure was unstable and variable, 140–105 – 125–80 mmHg. To abolish the symptoms of serotonin syndrome, the drug treatment of the time was withdrawn, and diazepam therapy was introduced [47, 48].

The simultaneous use of maprotiline and desmopressin may result in a general increase in water retention in the body, which may, in turn, lead to the development of the pathological condition of hyponatremia. In severe cases, this condition can result in life-threatening seizures, coma, or death [49]. Clinical case reports report an increased risk of dangerous bronchospasm with concomitant use of maprotiline, as well as phenelzine, nortriptyline, and desipramine. A 36 year-old man was treated with maprotiline 200 mg/24 h due to the occurrence of depressive episodes. The therapy carried therapeutic success. The man had no clinical history of respiratory problems or allergies present, but he developed dangerous breathing problems—wheezing, shortness of breath, and intractable and increasing respiratory distress. After taking bronchodilators, the adverse symptoms subsided. After maprotiline was experimentally excluded from the therapy at the time, the respiratory problems disappeared as the patient's eosinophilia levels decreased. When attempting to re-include maprotiline at a lower dose of 150 mg/24 h, the dangerous and bothersome respiratory symptoms returned. The patient received phenelzine, which resulted in therapeutic success without shortness of breath or breathing problems [50]. A single study suggests the occurrence of Oglivi syndrome, an acute pseudo-obstruction of the colon caused by maprotiline used in co-therapy with rosuvastatin and clopidogrel [51].

### Interactions of serotonin-norepinephrine reuptake inhibitors (SNRIs)

Selective serotonin and norepinephrine reuptake inhibitors SNRIs [52] mode of action involves a reuptake block of the neurotransmitters norepinephrine and serotonin – hence the reference to ‘dual-action’ drugs. The exact mechanism of action is based on the binding of norepinephrine transporter (NAT) and serotonin transporter SERT. They have no affinity for other receptors. Due to their nature, they cause an elevation of the overall level of the neurotransmitter dopamine in the prefrontal cortex [16]. These drug classes include venlafaxine, desvenlafaxine, milnacipran, duloxetine, and mirtazapine [53]. Duloxetine exhibits relatively unidirectional binding to both transporters, but the binding strength predominates toward serotonin. Venlafaxine shows significantly higher binding strength to the serotonin transporter than to the norepinephrine transporter, an effect that largely depends on the drug dose used. Milnacipran, on the other hand, shows the most equal affinity for both transporters compared to the drugs mentioned above [16]. Mirtazapine is characterized by its ability to raise neurotransmitters serotonin and norepinephrine levels. This effect occurs through an inhibitory effect on α-2 adrenergic heteroreceptors and autoreceptors. The drug also binds highly to histaminergic receptors [22].

The metabolism of duloxetine and mirtazapine mainly involves the cytochrome CYP2A1 subunit [26]. The drug may also act as an inhibitor of the cytochrome CYP2D6 subunit, which carries the risk of increasing the concentration in the body of drugs (Table 6). Also, medications from the SSRI group of selective serotonin reuptake inhibitors affect the concentration of duloxetine in the human organism. Paroxetine and fluvoxamine have been observed to raise the overall concentration of duloxetine in the body [16]. Studies show that mirtazapine is characterized by its ability to inhibit cytochrome CYP3A4, CYP2D6, and CYP1A2 subunits minimally.

**Table 6:** Interactions of antidepressants with SNRIs

Interactions of SNRIs with other drug classes			
Drug	Interacting substance	Results of interaction	References
SNRI	Benzodiazepines	Risk of impaired motor skills	[16, 22, 33, 54–63, 65, 67, 68]
	Calcineurin inhibitors Lithium inhibitors	Serotonin syndrome	
Duloxetine	Anticoagulant	Change in INR	
	Gastric juice antacids	Diarrhoea	
Venlafaxine	Propafenone	Visual and auditory hallucinations	
	Drugs used for weight reduction	Serotonin syndrome, tachycardia	
	Itraconazole Fluconazole Ketoconazole Mycoconazole	Increase in venlafaxine concentration	
	Apixaban, COX-2 inhibitors Clopidogrel Aspirin Warfarin Acenocoumarol	Increasing the risk of hemorrhagic complications and gastrointestinal bleeding	
	Amoxicillin and clavulanic acid Dihydroergotamine Preparations of common starwort Oxycodone Thistle Palonosetron Pethidine Selegiline Tramadol Triprolidine Trazodone Pentazocine Promethazine	Onset of serotonin syndrome	
	*Ginseng*	Hyperhidrosis, itching of the skin and also an increased risk of serotonin syndrome	
Mirtazapine	Clonidine	Hypertension	
	Phenothiazine	Increase of mirtazapine in the blood and subsequent side effects	
	Fluoxetine and *Rhodiola Rosea*	disturbance of consciousness	
	Levodopa	Occurrence of a psychotic state	
	Combination of sertraline and *Echinacea*	Serotonin syndrome	
	Ketoconazole Cimetidine Fluvoxamine	Increase of mirtazapine levels	
	Phenytoin Carbamazepine	Reduction of mirtazapine levels in the body	

Furthermore, there is a risk of impaired motor skills when this drug is used concomitantly with drugs from the benzodiazepine group [22]. In addition, it can exacerbate the sedative effects of medications with such properties as antipsychotics, alcohol, or benzodiazepines, which can lead to a significant increase in the risk of falls [16]. On the other hand, the CYP2D6 subunit and, to a slightly lower extent, CYP3A4 are involved in venlafaxine metabolism. Drugs in the group in question can also lead to serotonin syndrome when given concurrently with triptans or opiates. Also, venlafaxine used in simultaneous combination with compounds of calcineurin inhibitors, lithium inhibitors, tranylcypromine, or a combination of other SNRIs can lead to acute serotonin syndrome [54, 55].

Concomitant administration of duloxetine with anticoagulants also carries the risk of drug interactions [55]. A change in INR, the international normalized ratio, was observed as an effect of these interactions. There are many cases depicting this interaction. One of them is the case of a woman aged 63. The patient showed a decrease in her INR value when duloxetine 60 mg was added to her already existing acenocoumarol therapy. The value decreased from 2.54 to 1.49, and the index value at 1.49 persisted for three weeks since the duloxetine was discontinued. After this time, the INR value returned to normal [55]. Another case involves a woman aged 44 being treated with warfarin. After the inclusion of 30 mg of duloxetine in the current therapy, there was an increase in the INR value. The value rose even higher when warfarin withdrawal was applied. The index values decreased only when duloxetine exclusion from patient therapy was used [55]. Duloxetine may interact with gastric juice antacids, and the effect of this interaction may be the occurrence of diarrhea [56]. The literature also indicates the possibility of interaction between duloxetine and drugs metabolized by the cytochrome CYP2D6 subunit. Studies have shown that the simultaneous administration of desipramine and duloxetine leads to an increase in the Area under the curve (AUC) and maximum concentration (Cmax) values of desipramine. Because of the possibility of this phenomenon, particular caution, careful monitoring, and overall dose reduction of drugs such as tricyclic antidepressants or antiarrhythmic drugs (propafenone or flecainide) are recommended when used together with duloxetine [57]. Clinical cases also describe interactions between duloxetine and agomelatine administered concurrently. In a 62 year-old female patient treated with duloxetine hydrochloride, a significant increase in sweating was observed when agomelatine was added to her current therapy.

The occurrence of excessive drug-induced sweating was noted after treatment with agomelatine 25 mg and duloxetine hydrochloride 90 mg. The symptoms disappeared when agomelatine was discontinued, and trazodone was started [57]. Cases of drug-induced delirium have been reported with the combination of duloxetine and bupropion [58]. An 85 year-old man was treated with duloxetine at a dose of 60 mg/24 h. After seven days, bupropion was added to the therapy. It was observed that the patient was disoriented and could not recognize his place or the people with him. Additionally, there were visual and auditory delusions and persecution. These hallucinations occurred three days after bupropion was added to the therapy. It was decided to discontinue bupropion administration. After three days of discontinuing this drug from the patient’s treatment, the patient’s delusions and hallucinations gradually began to subside [58]. In addition, there is a risk of interaction when duloxetine is taken concomitantly with apixaban (increased risk of hemorrhagic complication syndrome, lower or upper gastrointestinal bleeding), brompheniramine (risk of dangerous serotonin syndrome), bupropion (cases of hallucinations and delirium), dihydroergotamine (risk of serotonin syndrome), encainide (a significant increase in the concentration of the drug in the body and subsequent side effects as a result of CYP 1A2 inhibition by duloxetine), ciprofloxacin (an enormously increased risk of increasing the concentration of duloxetine in the body—an absolute contraindication for combining the two drugs in pharmacotherapy), enoxacin (an increase in the concentration of duloxetine in the body based on CYP 1A2 inhibition) [33]. Furthermore, a marked increase in the risk syndrome of hemorrhagic complications and gastrointestinal bleeding was noted when duloxetine was co-administered with drugs such as corticosteroids, cyclooxygenase two inhibitors, aspirin, nonsteroidal anti-inflammatory drugs, rivaroxaban, ticlopidine, and warfarin [33].

The literature also indicates an increased possibility of stalling when venlafaxine is used concomitantly with drugs such as ibuprofen, aspirin, and naproxen [59, 60]. Cases of hallucinations and visual hallucinations can also occur when venlafaxine and propafenone are taken concurrently. Such symptoms were observed in a woman aged 85. The patient had been treated with propafenone 150 mg/12 h for three years. As a result of the occurrence of mood disorders, venlafaxine was included in the therapy at that time, initially at a dose of 75 mg/12 h. After one month, the dose of venlafaxine was raised to 150 mg/12 h. Ten days after increasing the dose of venlafaxine, the patient experienced significant psychomotor stimulation, hallucinations, and visual hallucinations. These symptoms worsened particularly at night. After four days of discontinuing venlafaxine, the unpleasant hallucinations ceased utterly [61]. In addition to the mentioned interactions, venlafaxine, when taken concurrently with cimetidine, can lead to the development of liver disease or hypertension. The literature also reports cases in which drugs such as clarithromycin, ketoconazole, or ritonavir that are co-administered with venlafaxine lead to an increase in venlafaxine concentrations in the body as a result of blocking its degradation. Also, the concomitant use of haloperidol with venlafaxine may lead to an interaction manifested as QT interval prolongation. It is also worth noting the risk of reducing the potency of metoprolol when taken together with venlafaxine [61]. There is also a risk of increased drowsiness and sedation when zolpidem or diphenhydramine is used concomitantly with venlafaxine [60]. The occurrence of interactions between venlafaxine and benzodiazepines is not entirely clear. Some sources mention the risk of increased sedation and tranquillity [60]. However, studies conducted with diazepam and venlafaxine show no significant pharmacodynamic or pharmacokinetic interactions [62]. J. Woroń et al. also report the possibility of interactions between venlafaxine and apixaban, COX-2 inhibitors, clopidogrel, aspirin, warfarin and acenocoumarol (increasing the risk of hemorrhagic complications and gastrointestinal bleeding), the combination of amoxicillin and clavulanic acid, dihydroergotamine, preparations of common starwort, oxycodone, thistle, palonosetron, pethidine, selegiline, tramadol, triprolidine, trazodone, pentazocine, and promethazine (onset of serotonin syndrome). In addition, combining therapy with ginseng can result in hyperhidrosis, itching of the skin, and also an increased risk of serotonin syndrome [63].

Clinical cases report the possible occurrence of significant hypertension with concomitant use of mirtazapine and clonidine. This effect occurred in a man in his 20 s who was treated with metoprolol, clonidine, and minoxidil. As a result of depression treatment, mirtazapine at a dose of 15 mg/24 h was included in his therapy. It has been noted that since taking mirtazapine treatment, the patient’s blood pressure began to rise rapidly to the point where it was no longer controlled as much as possible. After the patient was admitted to the intensive care unit and his blood pressure was regulated with nitroprusside, it was decided to discontinue mirtazapine treatment [64]. In addition, the occurrence of a possible interaction between benzodiazepines or alcohol and mirtazapine administered concurrently has been observed. Symptoms of such a combination include increased sedation and tranquilization [65]. No significant pharmacokinetic interactions were noted when mirtazapine was administered concurrently with lithium compounds, cimetidine, paroxetine, carbamazepine, amitriptyline, risperidone, and fluoxetine [65].

Additionally, there are single cases of the occurrence of interactions between mirtazapine and the combination of escitalopram and ginseng (the occurrence of serotonin syndrome), phenothiazine (increase of mirtazapine in the blood and subsequent side effects), combination with fluoxetine and Rhodiola Rosea (disturbance of consciousness), clonidine (mutual antagonization of the pharmacological profile of the drugs and subsequent risk of hypertensive breakthrough), combination therapy with clorazepate and ginseng (delirium along with excessive sedation), and levodopa (occurrence of a psychotic state) [63]. There are also cases of serotonin syndrome when mirtazapine therapy is combined with sertraline and Echinacea [63]. The study did not demonstrate a significant impact of food intake on metabolism and interactions with SNRIs [66]. A study suggests a possible increase in symptoms such as drowsiness, dizziness, motor coordination problems, impaired thinking, concentration problems, or confusion when taking duloxetine concurrently with valerian [67]. Another study suggests the risk of a pharmacokinetic interaction when venlafaxine is taken simultaneously with the Zuojin pill, which contains plant-derived substances such as Fructus Evodiae and Rhizoma Coptidis [68]. Other available scientific sources provide information on the possibility of hemorrhages and gastrointestinal bleeding during simultaneous intake of duloxetine with Ginkgo Bilobapreparations, incidents of anxiety and migraines, and headaches when taken with preparations containing thistle, incidents of intractable sore throat, jaundice, swallowing disorders, diarrhea, and hepatotoxicity when taken concurrently with Ginkgo preparations, as well as incidents of hyperhidrosis, pruritus, and risk of serotonin syndrome when combining duloxetine with ginseng [63].

### Interactions of norepinephrine reuptake inhibitors (NRIs)

Reboxetine belongs to a group of norepinephrine reuptake inhibitors used to treat depression [69]. It increases the concentration of norepinephrine in the synaptic gap by binding the NAT ant, which also blocks the extracellular reuptake of norepinephrine relative to the terminals [69]. The study showed that the use of reboxetine in the treatment of depression led to a reduction in the effects of chronic social stress on the neurotrophic brain-derived factor BDFN, the typical expression of the peroxisome proliferator-activated receptor PPAR-α, and the phosphorylated protein responsible for binding the cAMP-mediated response pCREB. With this activity, reboxetine has been proven to exhibit an additional antidepressant mechanism by promoting PPAR alpha expression in hippocampal structures [70]. The drug is mainly metabolized by the cytochrome CYP3A4 subunit [71]. Drugs that are inhibitors or inducers of this isoenzyme affect the metabolism of the drug in question (Table 7) [71]. Inhibitors of the CYP3A4 subunit can be divided into compounds with reversible and irreversible mechanisms. This type of inhibition can occur through several mechanisms—acting on the active site and covalently attaching an amino acid-derived residue at that site or through a mechanism based on alkylation or arylation of a heme-derived prosthetic group. The group of inhibitors also includes substances of plant origin [72]. The group of inducers of this subunit is much smaller [72].

**Table 7:** Interactions of NRIs with other medications drug classes and plant-derived agents

Interactions of NRIs with other drug classes			
Drug	Interacting substance	Results of interaction	References
Reboxetine	Enzyme CYP3A4 inhibitors: Troleandomycin Roxithromycin Oleandomycin N-desmethylerythromycin Irinotecan Erythromycin Clarithromycin Azithromycin 14-OH- clarithromycin Amiodarone Amlodipine Atazanavir Buprenorphine Clozapine Desipramine Diltiazem Diclofenac Felodipine Indinavir Isoniazid Levonorgestrel Lidocaine Midazolam Nicardipine Nortriptyline Phenelzine Ritonavir Sertraline Selegiline Tadaliphil Thiotepa, and verapamil Rutacarpin and limonin from Evodie fruit Gomisin from *Schisandra Chinesis* Bergamottin and its derivatives from the juice of Grapefruit Propranolone Dipyridamole Methadone and phenothiazine Enzyme CYP3A4 inductors: Rifabutin Rifampicin Carbamazepine Primidone Phenytoin Phenobarbital Clotrimazole Statins Cyclophosphamide Paclitaxel Ifosfamide	Increased plasma reboxetine concentrations, increased risk of bupropion side effects	[33, 71–75]
		Decrease in plasma reboxetine concentrations, increased risk of bupropion side effects	

Studies confirm the inhibitory effect of ketoconazole on reboxetine metabolism. After five days of simultaneous intake of reboxetine 4 mg and ketoconazole 200 mg, studies showed an increase in the AUC values for both enantiomers of reboxetine and a decrease in the clearance values for both compounds. An increase in the half-life of ketoconazole was also observed [73]. There are also cases of side effects related to the potentiation of noradrenergic effects with the simultaneous use of reboxetine with the synthetic hormone triiodothyronine T3. Studies have been conducted to determine the impact of administering the synthetic hormone as a potential adjunctive therapy for people struggling with advanced stages of depression. Side effects of this drug combination were observed in patients with symptoms such as irritability, increased body sweating, adverse urinary symptoms, sleep disturbances, tremors, and anxiety [22, 74]. Concomitant use of NRIs with reboxetine can cause the occurrence of sleep problems and insomnia. This effect may occur due to blocking the structure transporter for norepinephrine [75]. There is also a possibility of drug interactions occurring when reboxetine is taken simultaneously with amiodarone, propranolone, dipyridamole, methadone, and phenothiazine (displacement of both drugs from the junctions and subsequent increase in the free fraction of the drug in the patient’s body). There are also reports of a significant increase in the risk of hypokalemia during simultaneous pharmacotherapy with reboxetine and loop and thiazide diuretics [33]. As a result of the in vivo study proved that rats receiving a mixture of reboxetine—curcumin-containing extract showed significantly lower immobility values for chronic fatigue and forced swimming tests. In addition, animals in the groups receiving the mixture had significantly elevated levels of norepinephrine in brain structures. This study demonstrated the synergistic effect of the reboxetine-turmeric extract combination [76].

### Interactions of norepinephrine and dopamine reuptake inhibitors (NDRI)

Bupropion belongs to the group of selective norepinephrine and dopamine reuptake inhibitors [77]. The mechanism of action of this drug is based on blocking the reuptake of neurotransmitters such as dopamine and norepinephrine [22]. It blocks dopamine reuptake much more strongly compared to norepinephrine [78]. The drug also acts on nicotinic acetylcholine receptors, their non-competitive antagonist [79]. In addition, bupropion shows a weak affinity for receptors for serotonin [78]. It can reduce inflammatory cytokines like interferon-gamma or Tumour Necrosis Factor-alpha (TNF-alpha) [79].

The cytochrome CYP2B6 subunit mainly metabolizes the drug. It converts bupropion to hydroxybupropion with pharmacological activity [80]. Drugs that are inhibitors or inducers of this cytochrome subunit affect the metabolism of bupropion (Table 8) [80]. The inhibitor group of drugs causes a decrease in the enzymatic activity of the CYP2B6 subunit [80]. A reduction of 90% was reported with ticlopidine and 68% with clopidogrel [80].

**Table 8:** Interactions of NDRI with other medications drug classes

Interactions of NDRI with other drug classes			
Drug	Interacting substance	Results of interaction	References
Bupropion	Enzyme CYP2B6 inhibitors: Clopidogrel Ticlopidine Sertraline and thiotepa Orphenadrine Cyclophosphamide Paroxetine Clonazepam Fluvoxamine Norfluoxetine Nelfinavir, Diazepam and ritonavir Ketoconazole itraconazole	Increased plasma bupropion concentrations, increased risk of bupropion side effects	[22, 33, 80–83]
	Enzyme CYP2B6 inductors: Rifampicin Phenobarbital Corticosteroids and dexamethasone Carbamazepine Hyperforin Ritonavir Clotrimazole Efavirenz	Decreased plasma bupropion concentrations, impairment of the effects of bupropion	
	Flecainide	Elevation of flecainide concentration in the body entailing adverse side effects	
	Hormone replacement therapy	Significant elevation of bupropion concentration in the body and increased risk of seizures	
	Insulin	In case of hypoglycemia increased risk of convulsions	
	Linezolid	Risk of severe transient intraoperative pressure and dangerous risk of neurotoxicity	
	Moclobemide	Severe risk of neurotoxicity and bupropion intoxication	
	Metoprolol	Bradycardia, hypotension, severe sinus bradycardia, dizziness	

On the other hand, drugs that have inductors-properties can lead to increased metabolism and elevated levels of hydroxybupropion in the body [80]. Due to its ability to inhibit the cytochrome CYP2D6 subunit, bupropion is contraindicated for concomitant use with antipsychotics such as haloperidol, and other antidepressants such as sertraline, desipramine, group 1C antiarrhythmic drugs such as propafenone, flecainide and beta blockers like metoprolol [22]. The literature indicates an increased possibility of a seizure when bupropion is taken concomitantly with drugs that are characterized by their ability to lower the seizure threshold like tricyclic antidepressants and phenothiazines [22], tramadol, other drugs used to treat depression, drugs used for psychotic conditions, and theophylline [81]. Also, concomitant use with alcohol, lithium, glucocorticosteroids, olanzapine, and clozapine may lead to an increase in explosive effects on an additive basis by lowering the seizure threshold [33]. Bupropion should also be used with caution in combination with dopaminergic drugs due to its ability to enhance dopaminergic activity. Observations showed that the concomitant use of bupropion with levodopa used to treat Parkinson's disease increased patients' risk of nausea, hallucinations and delusions, confusion, agitation, and vomiting [22]. The simultaneous intake of levodopa-bupropion drug combinations can lead to a significant increase in the severity and frequency of levodopa side effects [82]. In addition, available scientific sources report that concomitant therapy with amantadine carries a high risk of patient neurotoxicity, and with memantine may exacerbate the risk of a seizure episode through a mechanism of cytochrome CYP 2B6 inhibition [33].

Monoamine oxidase inhibitors and bupropion used concurrently may pose a risk of interaction. This risk arises from the dual mechanism of action involving blocking monoamine reuptake and inhibiting their normal metabolism. Consequently, there's a potential for dangerous elevation of norepinephrine, serotonin, and dopamine levels in the body. To mitigate this risk, it is recommended to observe a minimum 14 day interval between discontinuing bupropion and initiating monoamine oxidase inhibitor therapy [81]. This break allows for restoring regular monoamine oxidase activity [81]. The case of a 57 year-old man presents the risk of combining bupropion with drugs that are mild reversible monoamine oxidase inhibitors with weak potency. As a result of a bypass graft infection, linezolid was included in the man's then-current therapy containing bupropion. After 24 h, the man developed severe hypertension with a blood pressure of 260/145 mmHg. Episodes of hypertension developed in the man due to the combination of bupropion and linezolid. In addition to its antimicrobial activity, linezolid is characterized by its ability to reversibly inhibit the enzyme low-potency monoxidase, which, combined with bupropion, manifested itself in episodes of hypertension [83]. For this reason, drugs belonging to the group of traditional monoamine oxidase inhibitors like tranylcypromine or phenelzine with bupropion have long been discontinued [83].

In addition to the cases described above, available scientific sources report the possibility of drug interactions when bupropion is taken concomitantly with efavirenz (the risk of lowering the concentration of bupropion in the body and lack of pharmacological effect preceding induction of CYP 2B6), flecainide (elevation of flecainide concentration in the body entailing adverse side effects, due to inhibition of cytochrome CYP 2D6), hormone replacement therapy (significant elevation of bupropion concentration in the body and increased risk of seizures), insulin (in case of hypoglycemia increased risk of convulsions), antifungal drugs such as ketconazole or itraconazole (induction of CYP 2B6 and increase of bupropion concentration in the body with risk of dangerous side effects), linezolid (concomitant use promotes risk of severe transient intraoperative pressure and dangerous risk of neurotoxicity), and moclobemide (severe risk of neurotoxicity and bupropion intoxication) [33]. Moreover, simultaneous intake with metoprolol can lead to cases of bradycardia, hypotension, severe sinus bradycardia, and also dizziness [33].

Also, substances of plant origin, such as baicalein, may affect the proper metabolism of bupropion. Through inhibition of the cytochrome CYP2D6 subunit, hydroxylation of bupropion was blocked. The study's results were analyzed by measuring the AUC field [84]. Also, plant extracts derived from ginkgo biloba may interfere with the normal metabolism of the drug in question. A study using recombinant liver-derived microsomal enzymes proved that extracts derived from this plant lead to blocking the hydroxylation of the antidepressant in question, which is related to the ability of Ginkgo-derived substances to inhibit CYP2D6 enzymes [85]. However, this effect needs to be clarified. There is another study conducted on men aged 19–25 who were given bupropion alone at a dose of 150 mg and in combination with a ginkgo preparation at a dose of 240 mg per day. The study was conducted for 14 days. Blood samples were collected from the subjects and evaluated for parameters of bupropion and its hydroxylated metabolite. A blood analysis showed that administration of the ginkgo preparation for 14 days did not significantly affect the pharmacokinetic parameters of hydroxybupropion or bupropion [86]. The literature also reports that substances from St. John’s wort herb can also interfere with the normal metabolism of bupropion [82].

### MAOIs interactions

Monoamine oxidase inhibitors (MAOIs) are medicines used to treat various forms of depression [87]. MAOIs affect by inhibiting the breakdown of these neurotransmitters, thereby enhancing their levels and allowing them to continue to act on depressed cells [88]. There are two kinds of monoamine oxidase: A and B. The monoamine oxidase A (MAO-A) is mainly spread in the liver, gut, and placenta, while monoamine oxidase A (MAO-B) occurs in the brain, platelets, and liver. Noradrenaline and serotonin are substrates of MAO-A, and methylhistamine, tryptamine, and phenylethylamine are substrates of MAO-B. Tyramine and dopamine are metabolized by MAO-A and -B [89]. MAOIs include moclobemide, phenelzine, isocarboxazid, selegiline, and tranylcypromine [90]. Due to side effects, several dietary restrictions, and safety concerns, MAOIs are not the drug of first choice for the treatment of mental health disorders. MAOIs can potentially cause many interactions: drug-food interaction, drug-to-drug interactions, and overdoses [91]. MAOIs should not be used with other antidepressants, such as SSRIs. A combination of these drugs can cause serotonin syndrome, which can be potentially fatal. What is more, when changing MAOIs to different antidepressants, patients should wait 14 days before starting the new treatment to prevent any drug interaction. MAOIs avert the breakdown of tyramine in the body and some foods, drinks, and other medications. When taking MAOIs and eating tyramine-containing foods, patients show high serum tyramine levels, leading to a sudden rise in blood pressure, known as the tyramine pressure response [92, 93]. Although it is rare, it can cause a cerebral hemorrhage, resulting even in death. [94]. Meperidine, tramadol, methadone, and dextromethorphan are contraindicated in patients taking MAOIs because they are at high risk of serotonin syndrome [90]. Taking moclobemide with omeprazole may result in a doubling of omeprazole levels and an increased risk of side effects [33]. Overall, SSRIs, TCAs, SNRIs, Serotonin antagonists and reuptake inhibitors (SARI), mirtazapine, bupropion, and sympathomimetic amines, including stimulants and St. John's Wort, are contraindicated with MAOIs [90].

### Interactions of drugs with receptor-based mechanisms of action

SARI works by inhibiting serotonin transporter and serotonin type 2 receptors. SARI blocks the alpha-1-adrenergic and histamine receptors and inhibits serotonin reuptake. SARI significantly induces a change in 5-HT presynaptic adrenoreceptors. However, the entire mechanism of action is not fully understood [94]. SARIs are inhibitors of the hepatic isoenzyme CYP3A4; therefore, when combined with drugs metabolized by this enzyme may increase the concentration of these drugs in the blood, increasing the risk of cardiotoxicity (Table 9) [18, 19, 23]. Administration of SARI with anxiolytic drugs, alprazolam, triazolam, and buspirone may improve their concentration in the blood [95]. The use of SARIs in combination with benzodiazepines is safe; however, increased soothing and sedative effects should be taken into account. Taking SARI drugs simultaneously with substances depressing the CNS, e.g., alcohol, can significantly intensify their effects [95].

**Table 9:** Interaction of drugs with receptor-based mechanisms of action drugs with other medications drug classes and stimulants

Interaction of drugs with receptor-based mechanisms of action with drugs			
Drug	Interacting substance	Results of interaction	References
Mianserin	Alcohol	Increase in sedative effects and hepatopathy	[18, 19, 23, 33, 95–99]
	Atomoxetine	Increase in seizure risk	
	Phenothiazine Clozapine Quetiapine Olanzapine TCAs	Summation of risk: sedation, myelotoxicity, hepatotoxicity	
	Carbamazepine	Increase in haematological and hepatological complications, attenuation of the effect of mianserin	
	NSAIDs	Possible decrease in analgesic effect due to central anticholinergic effect of mianserin	
SARI	Terfenadine Astemizole Pimozide	Risk of cardiotoxicity	
	Alprazolam Triazolam Digoxin Diphenylhydantoin	Increase concentration in the blood	
Trazodone	Buspirone Dextromethorphan Diphenhydramine Linezolid Pethidine Setrones Tramadol Tiprolidine Triptans	Serotonin syndrome	
	Omeprazole	*Mobitz type 1 heart block*	

What is more, taking other serotonergic medicines such as fentanyl, triptans, and TCA at the same time also increases serotonin levels [96]. Potential pharmacokinetic interaction of SARI drugs with digoxin and diphenylhydantoin has been identified, increasing serum concentrations of both drugs [97]. Nefazodone should not be used with TCAs because it increases their concentration in the blood [95]. Trazodone may also potentiate the effects of skeletal muscle relaxants and volatile anesthetics [98]. Trazodone also has a potential interaction with omeprazole. Trazodone may cause serotonin syndrome if taken together with buspirone, dextromethorphan, diphenhydramine, linezolid, pethidine, tramadol, triprolidine, and triptans [33].

Mianserin is referred to as an “atypical” antidepressant due to its unusual modes of action [99]. Mianserin acts as an antagonist of an α2-noradrenergic receptor. Moreover, it significantly exhibits antagonist properties at serotonin 5HT2A, 5HT2C, and 5HT3 receptors. It has also been noticed that mianserin increases dopamine and noradrenaline release in cortico-limbic areas of the brain [99]. The antidepressant effect of mianserin results from the blockade of presynaptic, hetero-, and auto-α2-adrenoceptors [100]. Interaction information is mainly derived from animal studies. It has been shown that caffeine and sildenafil increase the effect of MSR in the tail suspension test [101] and forced swim test [99]. This interaction is likely not pharmacokinetic. Studies on rats have shown that despite a moderate increase in blood pressure, mianserin does not affect the hypotensive effect of enalapril and propranolol and significantly increases the impact of prazosin [102].

There is also some indication that mianserin may intensify the antinociceptive effect of indomethacin and metamizole (but not morphine) in mice [103]. In addition, scientific sources report possible interactions of mianserin with alcohol (increase in sedative effects and hepatopathy), atomoxetine (increase in seizure risk), phenothiazine, clozapine, quetiapine, olanzapine, TCAs (summation of risk: sedation, myelotoxicity, hepatotoxicity), carbamazepine (increase in hematological and hepatological complications, attenuation of the effect of mianserin), NSAIDs (possible decrease in analgesic effect due to central anticholinergic effect of mianserin) [33].

### Interactions of drugs with other mechanisms of action

Agomelatine (AGT) is an agonist of the MT1 and MT2 melatonin receptors and an antagonist of 5-HT2C receptors. The psychotropic effect of agomelatine is due to the combination of its 5-hydroxytryptaminergic and melatonergic impact [104]. Agomelatine can modulate circadian rhythms. This property may be therapeutically crucial for an antidepressant, as depression is associated with circadian rhythm disruption [105]. The antidepressant effect of agomelatine has been proven in studies on depressed rodents [106]. The biotransformation is mainly mediated by CYP1A2 and, to a minor extent, by CYP2C9/19 [107]. Drugs that interact with CYP1A2 can significantly change the plasma levels of AGT (Table 10) [108]. Fluvoxamine, a strong CYP1A2 and moderate CYP2C9 inhibitor, substantially inhibits the metabolism of AGT, resulting in a sixfold increase in agomelatine exposure. A combination of estrogens with AGT (temperate CYP1A2 inhibitors) causes a several-fold increased exposure to AGT, though in experiments, this did not cause clinically relevant effects [82]. It has been shown that smoking induces CYP1A2 and decreases the bioavailability of AGT [82]. The use of AGT together with alcohol adds up to potential hepatotoxicity. During the coadministration of AGT with duloxetine, a case of akathisia [22, 82] and hyperhidrosis [82] have been reported. Both were classified as pharmacodynamic DDI with noradrenergic hyperstimulation. In the mouse depression model, caffeine strengthened the effects of agomelatine without changing its plasma levels [101]. In other studies on mice, hesperidin has been affirmed to potentiate the antidepressant effect of agomelatine [109].

**Table 10:** Interactions of drugs with other mechanisms of action with medications and plant-based agents

Interactions of drugs with other mechanisms of action			References
Drug	Interacting substance	Results of interaction	
Agomelatine	Enzyme CYP1A2 inhibitors: Fluvoxamine Amiodarone Ciprofloxacin Disulfiram Enoxacin Erythromycin Ketoconazole Clarithromycin Mexiletine Moclobemide Norfloxacin Ticlopidine	Significant increase in agomelatine concentration, potentiate its side effects	[22, 82, 108, 109], [3, 110, 112, 113, 117–120]
	Chlorpromazine Phenothiazines Carbamazepine Clozapine Milk thistle + sertraline Paracetamol Statins TCAs Alcohol	Potential hepatotoxicity	
	Phenobarbital Phenytoin Rifampicin Ritonavir Tipranavir	Reduction in agomelatine blood concentration	
	Duloxetine	Akathisia and hyperhidrosis	
Tianeptine	Salicylic acid	Increase in tianeptine concentration, potentiate its side effects	
Vilazodone	Ketoconazole	Increase in vilazodone concentration, potentiate its side effects	
Vortioxetine	Enzyme CYP2D6 inhibitors: Bupropion Fluconazole Ketoconazole Terbinafine Warfarin Hydrochlorothiazide Buspirone Dextromethorphan Diphenhydramine MAO inhibitors Linezolid Moclobemide Pethidine Antiemetic setrones SNRIs SSRIs TLDs Tramadol Trazodone at doses > 150 mg Triprolidine Triptans Phenytoin carbamazepine Rifampicin Piracetam	Increase vortioxetine concentrations and potentiate its side effects	
		Hemorrhagic complications	
		Hyponatremia	
		Increased risk of serotonin syndrome	
		Decrease vortioxetine concentrations and reduce its activity	
		Decreased elimination of piracetam and its increased concentration in the blood	

Tianeptine (TNP) is an antidepressant drug with a unique neurochemical profile. It increases serotonin uptake in the brain and reduces stress-induced atrophy of neuronal dendrites [110]. TNP is an agonist of the δ-opioid receptor (DOR) and the μ-opioid receptor (MOR). It is believed that MOR-agonism (or combined DOR/MOR-agonism) underlies the preclinical, clinical, and in vitro effects of TNP [111]. TNP binds strongly to albumin, which could theoretically lead to interactions with other drugs that bind to proteins, such as nonsteroidal anti-inflammatory drugs and sulfonylureas. However, due to the wide therapeutic margin, increased TNP concentrations due to the concomitant administration of these drugs are unlikely to be dangerous. TNP varies from most antidepressant medications because it is not mainly metabolized by the hepatic cytochrome P450, which indicates a lower probability of drug-drug interactions, which is especially important in elderly patients [110]. Alcohol administration decreased the TNP absorption rate and reduced plasma levels by around 30%; however, it did not influence the pharmacokinetics of the C5 metabolite. In studies evaluating the effect of antipsychotics, benzodiazepines, and many other drugs on the binding of TNP to plasma proteins, it was found that only salicylic acid in high plasma concentrations can displace TNP from its binding sites, which may increase its effect [112]. TNP significantly decreased the "wet dog shakes" induced by 5-hyroxytrytophan, which could reduce the probability of serotonin syndrome if tianeptine is inadvertently used with drugs that can cause serotonin syndrome [112]. In a mouse model of epilepsy, TNP increased the anticonvulsant effects of carbamazepine and valproate, but not phenytoin, without changing the concentration of these drugs in the brain. However, TNP reduced phenobarbital concentration in the brain but enhanced its anticonvulsant activity [113]. In mice, the activity of TNP was enhanced, presumably by increasing its brain concentration [114].

Vilazodone exhibits a dual mechanism of action. It acts as a potent and selective inhibitor of serotonin 5-HT reuptake and selectively binds to 5-HT1A receptors with high affinity [115]. Functioning as both an SRI and a 5-HT1A partial agonist, vilazodone undergoes metabolism primarily via CYP3A4, with additional metabolism by CYP2C19 and CYP2D6, as well as possibly through non-CYP pathways such as carboxylesterases [116]. Simultaneous administration of vilazodone with a proton pump inhibitor or ethanol does not extend or affect the rate of vilazodone absorption. Consequently, when combined with potent CYP3A4 inhibitors, vilazodone dosage should be reduced to 20 mg. No dose adjustment is recommended for mild CYP3A4 inhibitors. CYP3A4 inducers are expected to minimize vilazodone concentration, which may decrease the effectiveness of the drug.

Minimal effects on vilazodone have been noted with other CYP isoenzyme inhibitors and inducers. Research in healthy volunteers revealed that vilazodone at a dosage of 20 mg/day administered for 8–10 days with flurbiprofen, nifedipine, caffeine, or debrisoquine, probes for CYP2C9, CYP3A4, CYP1A2, and CYP2D6, respectively—did not significantly affect their pharmacokinetic profiles. Additionally, in vitro experiments suggest that vilazodone may inhibit biotransformation of CYP2C8 substrates. Simultaneous administration of vilazodone with any drug that affects serotonergic neurotransmitter systems ought to be carefully applied, and it needs clinical monitoring because of the potential for serotonin syndrome [117]. Vortioxetine is a novel antidepressant for the treatment of major depression [118]. This drug is metabolized by many enzymes [119]. CYP2D6 is an enzyme that mainly metabolizes vortioxetine; CYP2C19 plays a minor role in this process. There are known interactions between vortioxetine and other drugs. Active metabolites of bupropion-threohydrobupropion and erythrohydrobupropion- are potent inhibitors of CYP2D6. Occurrences of side effects, such as nausea, vomiting, headache, and insomnia, were three times more likely after adding bupropion to vortioxetine monotherapy as when vortioxetine was added to bupropion therapy or when vortioxetine was used without other drugs [119]. Lots of CYP enzymes are induced by rifampicin, for example, CYP3A4/5, CYP2C19, CYP2C9, CYP2C8, and CYP2B6. Because of this, it is recommended to take into consideration increasing the dose of vortioxetine during treatment with rifampicin or other potent CYP inducer, for example, phenytoin or carbamazepine, when they are administered more than 14 days [119].

In the study of Woroń et al., there are described two cases of hemorrhagic complications—nasal and vaginal bleeding-after a combination of vortioxetine and warfarin. Another case concerns hyponatremia, which occurred as a result of a combination of vortioxetine and hydrochlorothiazide [3]. Available evidence indicates that vortioxetine does not significantly affect the pharmacokinetics of warfarin or increase its concentration [119]. The observed bleeding events may be linked to vortioxetine's mechanism of action, which involves blocking serotonin reuptake on platelets, similar to SSRIs and SNRIs [120]. It is presumed that the mechanism underlying hyponatremia may parallel that of SSRIs, involving the risk of hyponatremia due to diuretic action and the potential induction of syndrome of inappropriate antidiuretic hormone secretion associated with serotonin reuptake blockade [3].

### New drug interactions – ifenprodil

Ifenprodil is a new drug from the anti-n-methyl-d-aspartate receptor (NMDAR) receptor antagonist group that has found use as an innovative antidepressant in recent years [121]. The mechanism of action of this drug is based on blocking G-protein-stimulated potassium channels [122]. Additionally, it blocks the previously mentioned n-methyl-d-aspartate receptor (NMDA) receptors through a conservative pathway by affecting GluN subunits [121]. The study, conducted with a mouse model of anxiety disorders, showed a change in the behavioral outcomes of the test animals in the track of simultaneous administration of ifenprodil together with dizocilpine. During the conducted experiment, the researchers noted a significant ability to suppress the deficit caused by the administration of dizocilpine, whereas, during the isolated administration of ifenprodil, the described effect did not occur. Both drugs were administered at a dose of 10 ml/kg (0.25 ml of the drug) via the intraperitoneal route. Mice evaluated during the maze plus test showed the effect of ifenprodil combined with dizocilpine—the addition of ifenprodil to the therapy induced a decrease in anti-anxiety effects and a decrease in the overall locomotor activity of dizocilpine. The experiment also noted the absence of degenerative effects of ifenprodil on working memory, with the simultaneous presence of such effects for dizocilpine [122, 123].

In another study involving mice, a synergistic antidepressant-like effect was observed with the combination of lithium and ifenprodil treatment. The test substances were administered to animals at a dose of 5 ml/kg (lithium) and 0.1/0.5/3 mg/kg (ifenprodil) of mouse body weight via the intraperitoneal route. The injections were performed 30 (lithium) and 45 (ifenprodil) minutes before the selected test. The combination of both drugs resulted in a significant enhancement of the antidepressant effect compared to the control group, as assessed by the forced swim test [124]. Single available publications also present the enhancing effect of ifenprodil when combined in pharmacotherapy together with paroxetine. In an experiment using a mouse model of depression, they showed a beneficial effect of the paroxetine-ifenprodil combination, characterized by an enhanced antidepressant effect [125].

### Interactions of drugs with adaptogens

Recently, many patients suffering from depression and mood disorders often associated with the disease have been turning to so-called “adaptogens” Adaptogens are non-toxic substances of plant origin that increase “non-specific” resistance to adverse biological, chemical, and physical factors, normalizing body functions and enhancing stress resistance [126]. Because the principle of adaptogenic action has yet to be precisely explained, this term is currently not accepted in clinical and pharmacological terminology in the European Union; however, it is now widely used, sometimes even by doctors and pharmacists. However, there are scientific reports that taking adaptogens is associated with a better ability of the body to adapt to stress and normalize metabolic functions, as well as with better mental and physical performance [127–129]. Many reports suggest that adaptogens may improve sleep, alleviate fatigue, anxiety, memory impairment, and depressive symptoms, and reduce stress levels [130]. Therefore, in recent years, there has been a steady increase in interest in adaptogens among patients suffering from depressive disorders, who take them in addition to medications prescribed by doctors as a form of complementary treatment or to alleviate side effects occurring during psychopharmacotherapy [131]. As a rule, these substances are non-toxic and usually well tolerated, but they may cause adverse interactions with concurrently taken antidepressants.

Interestingly, research conducted by Woroń et al. showed that the frequency of interactions of “adaptogens” with antidepressants was twice as high as the occurrence of adverse events caused by interactions of antidepressants with over-the-counter drugs [132]. As the authors of the study note, in clinical practice, the incidence of these interactions may be much higher. It is worth emphasizing that dietary supplements referred to as adaptogens most often contain plant extracts consisting of many separate, pharmacologically active substances, which additionally significantly increases the risk of adverse events [63]. Woroń et al. performed a retrospective review assessing the incidence and clinical characteristics of adverse events associated with the concomitant use of antidepressants and adaptogens [131]. They evaluated 326 reports and found that 9% of adverse events due to antidepressant drug interactions were due to the concomitant use of adaptogens, especially *Withania somnifera* (Ashwagandha), *Eleutherococcus senticosus, Schisandra chinensis, Tribulus terrestris, Coptis chinensis, Cimcifuga racemosa, Bacopa monnieri, Gynostema pentaphyllum, Cordyceps sinensis, Lepidium meyenii,* and *Scutellaria baicalensis.* The undesirable effects of the interaction of adaptogens with antidepressants are presented in Table 11. The hypothesis put forward by the authors of the study may be confirmed by the fact that in all cases analyzed, discontinuation of adaptogen supplements led to remission of the side effects. The main mechanisms responsible for the occurrence of unfavorable phenomena when taking antidepressants and adaptogens simultaneously include interactions with cytochrome p-450 and P-glycoprotein. The main mechanisms responsible for the development of unfavorable phenomena when taking antidepressants and adaptogens simultaneously include interactions with cytochrome p-450 and P-glycoprotein, in particular, inhibition of isoenzymes involved in drug metabolism and inhibition of transport proteins. As a result of these interactions, many side effects may occur, in some cases life-threatening, such as bleeding from the upper gastrointestinal tract or myocardial infarction.

**Table 11:** Interactions of drugs with adaptogens

Interactions of drugs with adaptogens			References
Adaptogen	Interacting substance	Result of interaction	
*Withania somnifera*	Reboxetine	Testicle pain and ejaculatory dysfunctions	[63, 131, 132]
	Sertraline	Severe diarrhea	
	Escitalopram	Myalgia, epigastric pain, nausea, vomiting, restless legs syndrome, and severe cough	
	Paroxetine	Generalized myalgia, ophthalmalgia, and ocular hypertension	
*Eleutherococcus senticosus*	Duloxetine	Upper gastrointestinal bleeding	
	Paroxetine	Epistaxis	
	Sertraline	Vaginal hemorrhage	
	Agomelatine	Irritability, agitation, headache, and dizziness	
*Schisandra chinensis*	Bupropion	Arthralgia and thrombocytopenia	
	Amitriptyline	Delirium	
	Fluoxetine	Dysuria	
*Tribulus terrestris*	Citalopram	Generalized pruritus	
	Escitalopram	Galactorrhea	
	Trazodone	Psoriasis relapse	
*Coptis chinensis*	Mianserin	Arrhythmias	
	Mirtazapine	Edema of lower limbs and myalgia	
	Fluoxetine	Gynecomastia	
*Cimicifuga racemosa*	Mianserin	Restless legs syndrome	
	Paroxetine	Gynecomastia and mastalgia	
	Venlafaxine	Hyponatremia	
*Bacopa monnieri*	Agomelatine	Back pain and hyperhidrosis	
	Moclobemide	Myocardial infarction	
*Gynostemma pentaphyllum*	Duloxetine	Back pain	
*Cordyceps sinensis*	Sertraline	Upper gastrointestinal bleeding	
*Lepidium meyenii*	Mianserin	Restless legs syndrome	
*Scutellaria baicalensis*:	Bupropion	Seizures	
*Japanese ginkgo biloba*	SSRI or	Hemorrhagic complications	
	SNRI		
*Panax ginseng*	Haloperidol	Ventricular arrhythmias, pancreatitis	
	Risperidone	Pancreatitis	
	Aripiprazole	Hepatotoxicity	

### Interactions of drugs with CBD

Patients suffering from depression, often on their own, without consulting a doctor, use cannabidiol (CBD), for which scientific data on safety and effectiveness in the treatment of depression are limited and inconclusive. CBD is a natural compound found in cannabis, whose therapeutic effects are currently widely advertised. However, the FDA (Food and Drug Administration) has concluded that “*The use of CBD raises various safety concerns, especially with long-term use”* [133]. Studies have shown that CBD has potentially harmful effects and the possibility of severe negative drug interactions—the FDA-approved epilepsy drug (Epidiolex) has risks of liver damage, excessive sedation, suicidal thoughts, and hypersensitivity reactions [133]. Studies have also shown potential effects such as chronic psychosis and anxiety. Nowadays, the use of CBD, a phytocannabinoid derived from cannabis that does not have intoxicating properties, is becoming more and more popular. Unfortunately, this compound is not without risk of interaction with other substances, including antidepressants. Available studies show that CBD is metabolized by cytochrome CYP450 isoenzymes, including CYP2D6, CYP2C19, CyP3A4, CYP1A2, and CYP2C9, on which it has an inhibitory effect. This action may increase the concentration of SSRI and TCA drugs. In addition, CBD interacts with MAOIs by inhibiting their metabolism, which may increase side effects [134, 135].

## Conclusions and future perspectives

Injudicious prescription of antidepressants to patients suffering from other diseases may cause unfavorable drug interactions, the most severe effects of which include gastrointestinal bleeding, cardiovascular disorders, bradycardia, serotonin syndrome, neuroleptic syndrome, hepatotoxicity, increased risk of seizures, and in some cases – even death. Therefore, pharmacotherapy of patients with depression and comorbidities should be balanced, preceded by a detailed analysis of the safety assessment, followed by the search for the safest alternative drug combinations. There is a need for monitoring and reporting of adverse events resulting from drug interactions, as well as in-depth education in this area. At the same time, medical practitioners should keep in mind the undesirable interactions are occurring in patients who, in the treatment process of other diseases, self-medicate depression with so-called adaptogens. Such cases unveil the significance of a thorough medical interview regarding prescription drugs and more and more commonly used dietary supplements.

## Data Availability

No datasets were generated or analyzed in the current study.
